# Metabolic Syndrome Among Primary Health Care Nursing Professionals: A Cross-Sectional Population-Based Study †

**DOI:** 10.3390/ijerph16152686

**Published:** 2019-07-27

**Authors:** Magno Conceição das Merces, Amália Ivine Costa Santana, Iracema Lua, Dandara Almeida Reis da Silva, Douglas de Souza e Silva, Antonio Marcos Tosoli Gomes, Manuela Conceição das Merces Miranda, Caroline da Silva Barbosa, Lucélia Batista Neves Cunha Magalhães, Julita Maria Freitas Coelho, Maria Lucia Silva Servo, Daniel Deivson Alves Portella, Marcio Costa de Souza, Sueli Bonfim Lago, Edilene Maria Queiroz Araújo, Sergio Correa Marques, Virgínia Paiva Figueiredo, Argemiro D’Oliveira Júnior

**Affiliations:** 1 Department of Life Sciences, State University of Bahia (UNEB), Salvador 41150-000, Brazil; 2 Health Sciences Postgraduate Program, School of Medicine, Federal University of Bahia (UFBA), Salvador 40026-010, Bahia, Brazil; 3 Department of Health, State University of Feira de Santana (UEFS), Feira de Santana 44036-900, Brazil; 4 School of Nursing, State University of Rio de Janeiro (UERJ), Rio de Janeiro 20551-030, Brazil; 5 Department of Family Health, School of Medicine, Federal University of Bahia (UFBA), Salvador 40026-010, Bahia, Brazil

**Keywords:** metabolic syndrome, nursing, primary health care, work

## Abstract

This research aims at evaluating prevalence and factors associated with metabolic syndrome (MS) in primary health care (PHC) nursing professionals. A multicenter, population-based and cross-sectional study was conducted in a team-tested sample of 1125 PHC nurses in the state of Bahia, Brazil. Sociodemographic, labor, lifestyle and human biology variables were investigated by mean of anamnesis. MS was evaluated according to the criteria of the first Brazilian Guideline for Metabolic Syndrome, which fully adopts the criteria of the National Cholesterol Education Program’s Adult Treatment Panel III. MS-associated factors were tested by using robust Poisson Regression. The prevalence of MS found was 24.4%; low High Density Lipoprotein (HDL) cholesterol was the most prevalent component of the syndrome. In the multivariate analysis, physical inactivity (PR = 1.25, 95% CI = 1.02–1.53), alcohol use (PR = 1.84, 95% CI = 1.22–2.77), acanthosis nigricans (PR = 3.23, 95% CI = 2.65–3.92), burnout syndrome (PR = 1.45, 95% CI = 1.17–1.81), (PR = 1.37, 95% CI = 1.12–1.69), working as a nursing technician (PR = 1.43, 95% CI = 1.14–1.80), were associated to MS. It was found that the prevalence of MS was high, which evidences the need for interventions in the PHC environment, improvement of working conditions, monitoring of worker safety and health, diet programs and physical activity.

## 1. Introduction

After the Industrial Revolution, massive transformations imposed on society established certain morbidity and mortality profiles. Because of a mismatch among biological factors, which selected energy-saving individuals with sedentary habits due to technological development, there was an imbalance between consumption and energy expenditure, which, eventually, ended up increasing metabolic diseases around the world [1].

In this context, we highlight metabolic syndrome (MS), which is a clinical condition with several cardiovascular risk factors. Due to its risks, this syndrome has been increasingly investigated. The syndrome presents a two-fold increased risk for cardiovascular diseases and one-and-a-half times greater risk for all-cause mortality [2]. Although the syndrome etiology is not fully elucidated, it is defined by the combination of at least three of the following five parameters: abdominal obesity, triglycerides, High Density Lipoprotein (HDL) cholesterol, blood pressure, and fasting blood glucose in accordance with the National Cholesterol Education Program’s Adult Treatment Panel III (NCEP-ATP III) [3]. Several factors are pointed out as increasing exposure to MS: polycystic ovary syndrome [4], periodontitis [5], burnout syndrome [6], acanthosis nigricans [7], working conditions [8].

Consistent scientific evidence has not yet been established to prove the direct relationship between MS and labor activity. However, it is assumed that working conditions can develop this syndrome due to what workers are submitted and exposed to, namely: erroneous nutritional habits caused by irregular eating schedules, night and shift work, lack of time for leisure and caring of one’s own health, among others [9].

Regarding primary health care (PHC), the impact of working conditions on nursing teams and other professionals can be a source of illness by placing workers in environments without infrastructure, low salaries, precarious ties, care service overload, absenteeism, presenteism, among others. Thus, these professionals experience a progressive wear that compromises their health and the quality of care provided.

In the specialized literature, no scientific investigations were proposed to elucidate MS occurrence in the referred population. This research aims at evaluating prevalence and factors associated with metabolic syndrome (MS) in primary health care (PHC) nursing professionals in the state of Bahia, Brazil.

## 2. Materials and Methods

### 2.1. Study Design, Site and Sample

This is a cross-sectional and exploratory study, based on data from a multicenter population-based epidemiological survey, including a representative sample of PHC nursing professionals from Bahia state, Brazil, selected by cluster-based sampling and stratified by mesoregions.

**Figure 1:**
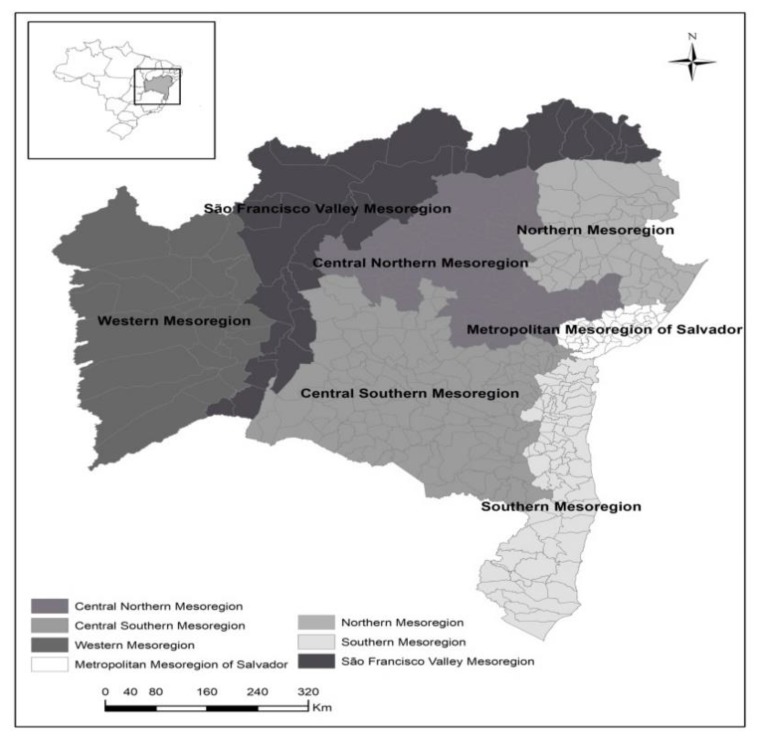
Distribution of mesoregions, Bahia, Brazil, 2017.

The state of Bahia consists of 417 municipalities, organized in seven mesoregions, namely: Western Mesoregion (24); São Francisco Valley Mesoregion (27); Central Northern Mesoregion (80); Northern Mesoregion (60); Metropolitan Mesoregion of Salvador (38); Central Southern Mesoregion (118) and South Mesoregion (70) (Figure 1) [10].

**Figure 2:**
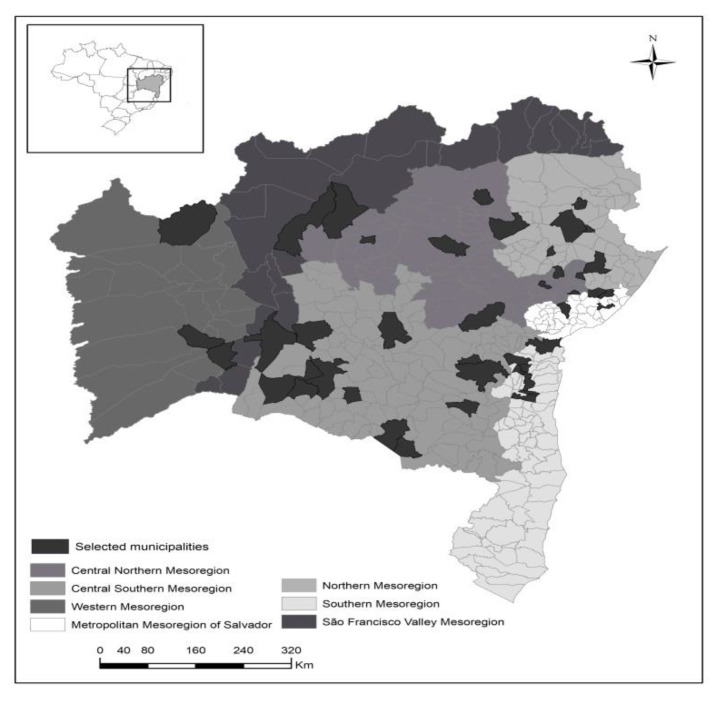
Distribution of eligible municipalities, Bahia, Brazil, 2017.

Ten percent of the municipalities (cluster-based) of each stratum (mesoregion) were drawn using the program Microsoft Office Excel version 2010, totaling 43 municipalities. We included all PHC nursing professionals of the conglomerates drawn, totaling 1195 individuals (Figure 2).

Epi Info 7.0 (Centers for Disease Control and Prevention, Atlanta, GA, USA) was used to calculate the sample size. Since no studies were found to indicate the MS rate in the exposed and unexposed group, a pilot study was carried out in a similar population. The MS rate is 20% and 33.3% in the non-exposed and exposed groups, respectively, where there is an error *α* of 0.05, power of 90% (1—error *β*), ratio 1:1, reaching a sample n of 464. The design effect of 2.0 (sample by clusters) was also considered, the sample was doubled to 928, and 20% was added to the possible losses and refusals. Therefore, 1114 PHC nursing professionals were obtained in total.

### 2.2. Eligibility Criteria

Forty-eight PHC nursing professionals were excluded because of the following: medical leave, less than 6 months of experience in PHC, solely administrative activities, pregnant women, menstrual period, depression diagnosed, anxiety and burnout before taking the job position, liver cirrhosis, and alcohol and drug dependence. Twenty-two PHC nursing professionals declined to participate in the research. The response rate was 94.1%.

### 2.3. Data Collection and Variable Definition Procedures

Anamnesis and data collection were performed in health care units between 2017 and 2018, by using a record sheet previously tested and containing socio-demographic variables regarding labor, lifestyle and human biology. To guarantee homogeneity in the application of the record sheets, a calibration was carried out among the research assistants, by interviewing thirty hospital professionals. The concordance among the assistants was calculated by using the Kappa index. A value of 0.87 was found and considered acceptable [11].

The dependent variable studied was MS, considered as a yes or no for the analysis. A diagnosis of MS was defined when three or more factors were presented according to the first Brazilian Guideline for Treating and Diagnosing Metabolic Syndrome [12], which fully adopts the NCEP-ATP III [13]. The possible factors were: (1) abdominal obesity based on abdominal circumference ≥ 102 cm (male) and ≥ 88 cm (female); (2) triglycerides ≥ 150 mg/dL, or use of medications for dyslipidemia; (3) high density lipoprotein (HDL cholesterol) < 40 mg/dL (male) and <50 mg/dL (female), or use of medications for dyslipidemia; (4) systolic pressure ≥ 130 mmHg or diastolic pressure ≥ 85 mmHg, or use of antihypertensives; (5) fasting glycemia ≥ 110 mg/dL or previous diagnosis of diabetes [12,13].

The most representative and the simplest anthropometric check of intra-abdominal fat is the waist circumference (WC) due to its deep relation with the amount of visceral adipose tissue and it is the best indicator of visceral fat mass [14]. The WC was measured two times and at the midpoint of the horizontal distance between the lower border of the costal grid and the iliac, at orthostatic position, arms along the body, feet together, weight divided between the legs and the face in straight position [15]. An inelastic and glassed measuring tape was used, divided into 0.1 cm, ISP^®^ brand (Wiso, Santa Tereza, Paraná, Brazil). Standardization and assessment of WC followed the recommendations of the Nutrition Department of the University of São Paulo, Brazil.

The blood samples were obtained after a 12-h fast and then analyzed in a reference laboratory at each municipality studied. For the serum levels of fasting glycemia, HDL cholesterol and triglycerides, conventional enzymatic and colorimetric laboratory techniques were used. An insulin fasting test was carried out by chemiluminescence. Blood pressure was measured by using a stethoscope (Littmann^®^, Classic III, 3M, USA)) and an aneroid sphygmomanometer (BD^®^ adult medium size, USA), previously calibrated. Two measurements were made on the left upper limb of the nursing professional, after five minutes of rest. The average value considered was taken between two measurements within 5 min.

As for sociodemographic and labor aspects, the independent variables were the following: sex (male = 0/female = 1); age (up to 35 years = 0/36 years or older = 1); profession (Nurse = 0/Nursing Technician = 1); race/color (not black = 0/black = 1); satisfaction with the current occupation (yes = 0/no = 1); occupation time in PHC (up to 4 years = 0/5 years or more = 1); night shift (no = 0/yes = 1); family income (up to two minimum wages = 1/3 or more minimum wages = 0); economic situation (satisfied = 0/unsatisfied = 1), work bond (stable = 0/precarious = 1); submitted to work-related aggression (no = 0/yes = 1); rest break (yes = 0/no = 1); working conditions (satisfactory = 0/precarious = 1). The variables described were self-reported.

Regarding lifestyle and human biology, the independent variables were the following: quality of life/ self-reported(good = 0/bad = 1); polycystic ovary/ self-reported (no = 0/yes = 1); psychiatric follow-up/ self-reported (no = 0/yes = 1); practice of physical activities/ self-reported (yes = 0/no = 1); smoking/ self-reported (not = 0/yes = 1); consumption of alcoholic beverage/ self-reported (no = 0/yes = 1); last medical consultation/ self-reported (less than 12 months = 0/more than 12 months = 1); presence of periodontitis/ self-reported (no = 0/yes = 1); acanthosis nigricans (no = 0/yes = 1); non-alcoholic fatty liver disease/ self-reported (no = 0/yes = 1); obstructive sleep apnea/ self-reported (no = 0/yes = 1); insulin resistance (no = 0/yes = 1); burnout syndrome (no = 0/yes = 1).

In order to identify acanthosis nigricans, the cervical region was evaluated, followed by the armpits, limb flexor surfaces, as well as the periumbilical and inframammary regions. Insulin resistance was measured by the Homeostasis Model Assessment-Insulin Resistance (HOMA-IR) index, obtained by calculating the fasting plasma insulin (μU/mL) and fasting glycemia (mmol/L) divided by 22.5 [16]. As a cut-off point, HOMA-IR > 4.65 [17] was adopted.

Burnout syndrome was evaluated by the Maslach Burnout Inventory-Human Services Survey [18], a version adapted and validated in Brazilian Portuguese by Tamayo [19]. Burnout syndrome was dichotomized according to the criterion of Ramirez et al. [20] as present or absent, based on high scores for the dimensions of emotional exhaustion and depersonalization and low scores on reduced professional achievement.

### 2.4. Data Entry

Data typing and processing were performed on the Statistic Package for Social Sciences—SPSS, version 22.0 (IBM Corporation, Nova York, NY, USA) for Windows. Data analysis were made on STATA for Windows, version 14.0 (StataCorp, College Station, TX, USA), in the Laboratory for Teaching, Research and Extension in Collective Health (LEPESC) of the State University of Bahia (UNEB), Brazil.

### 2.5. Statistical Analyses

A descriptive analysis was used for characterizing the sample and estimating the prevalence of the outcome. This analysis was expressed in tables at absolute and relative frequencies. By using ArcGIS 10.3 software (ESRI Inc., Redlands, CA, USA)), a map with the MS prevalence was developed per mesoregion on Geographic Information System (GIS) environment.

Then, a bivariate analysis was performed to evaluate the gross association between independent and dependent variables (MS) by using prevalence ratios (PR), their 95% confidence intervals (CI). In addition, a significance level of 5% (*p*-value ≤ 0.05) based on the Pearson Chi-square test was used to analyze the statistical significance of the associations found and the selection for the next step.

For a multivariate analysis, the backward logistic regression was used based on associations with *p* value ≤ 0.25. After that, it was possible to estimate the MS-associated factors by using a selection criterion of *p* ≤ 0.05 for the variable permanence in the final model. The PR and its CI were obtained through Poisson robust regression, a method also used by Coutinho, Scazufca and Menezes [21], Francisco et al. [22] for odds ratio (OR) conversion (obtained from logistic regression models) in PR.

The final regression model adequacy was based on Hosmer and Lemeshow’s godness of fit and the area under the Receiver Operating Characteristic (ROC) curve.

### 2.6. Ethical Aspects

The study was approved by the Research Ethics Committee involving human beings of State University of Bahia (UNEB), Brazil, opinion 872.365/2014. The guidelines of the Helsinki Declaration were followed.

## 3. Results

**Figure 3:**
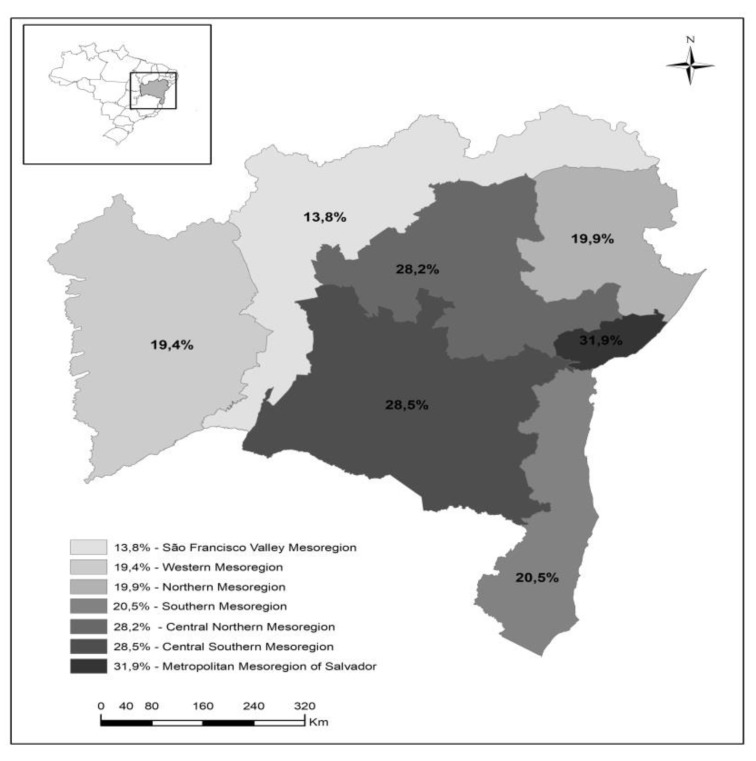
Prevalence of metabolic syndrome in nursing professionals of primary health care distributed per mesoregions of Bahia, Brazil, 2018.

The study population consisted of 1125 nursing professionals, who are young (mean = 37.1 years, SD ± 9.6), black and mostly female. The overall prevalence of MS was 24.4% with a diverse spatial distribution ranging from 13.8% to 31.9% (Figure 3). The MS features were the following: HDL cholesterol reduction (44.0%), increase in waist circumference (41.5%) and hypertriglyceridemia (33.4%).

The prevalence of the outcome had a statistically significant association with age greater than 36 years (PR = 1.59, 95% CI = 1.29–1.97), high school level profession (PR = 1.64, 95% CI = 1.29–2.07), black race (PR = 1.35, 95% CI = 1.02–1.78), occupation time in PHC greater than 5 years (PR = 1.47, 95% CI = 1.19 (OR = 1.27), income up to 2 minimum wages (PR = 1.27, 95% CI = 1.03–1.56), submitted to work-related aggression (PR = 1.24, 95% CI = 1.01–1.53) and without rest breaks (PR = 1.30, 95% CI = 1.06–1.59) (Table 1).

Habits of life were found to be positively associated to the occurrence of MS, especially the use of alcoholic beverages (PR = 1.64, 95% CI = 1.10–2.44). Regarding clinical conditions, acanthosis nigricans (PR = 3.52, 95% CI 2.92–4.24) and insulin resistance (PR = 3.55, 95% CI = 2.09–6.04) increased by more than three times the outcome in the study population, both with statistical significance (Table 2).

The multivariate analysis allowed us to establish the final model of associated factors. The variables that remained were sociodemographic features, life habits and human biology. Among such variables, most are strongly associated with the acanthosis nigricans outcome (PR = 3.23, 95% CI = 2.65–3.92). On the other hand, physical activity had a weaker association (PR = 1.25, 95% CI = 1.02–1.53). The goodness-of-fit test (>0.05) and the area under the ROC curve (0.70) showed that the model fit satisfactorily to the data, presented adequate discriminating power and, therefore, a reasonable predictive capacity (Table 3).

## 4. Discussion

This is the first Brazilian study to investigate factors associated with MS in PHC nursing professionals. After multivariate analysis it was found that lack of physical activity, alcohol consumption, acanthosis nigricans, burnout syndrome, age greater than 36 years and being a nursing technician are able to predict MS in the scenario studied. However, acanthosis nigricans is the most associated and discriminating marker in comparison to other variables.

Little is known about the prevalence of MS in the labor force. No studies were found with the object proposed here, and it is not possible to compare the results presented with a similar population. There are few investigations seeking to know the factors associated with MS in workers in general [23,24].

When assessing the prevalence and factors associated with MS among 4666 industry workers in Taiwan, China, based on the criteria of the Taiwan National Health Department for the MS diagnosis, Tsai; Cheng; Lai [23] showed a prevalence of 8.2%, higher among men (14%). In addition, they showed that waist circumference, systemic arterial hypertension (SAH) and hypertriglyceridemia were the most common MS marker. Being older was also correlated to MS. Felipe-de-Melo et al. [24] analyzed the factors associated with MS among 1387 administrative workers from the oil industry in Salvador, Brazil, and they found a prevalence of 15%, higher among men (74.6%), based on NCEP-ATP III criteria [13] and I-DBSM [12]. Blood glucose level was a frequent factor, followed by obesity and SAH. Being male, 40 years old or older, and a past or current smoker are associated with MS.

These findings are in line with this study, since a higher prevalence of MS among men was observed (29.4%), even though female population was almost eight times higher. Some likely explanations to this fact are less self-care awareness in men, a tendency to accumulate visceral fat deposits as age increases, and the fact that this study was conducted with young women, who have higher estrogen levels and, thus, more cardiovascular protection [23,25,26]. Nursing professionals over 36 years old are a critical factor for MS. This result is similar to other investigations [8,27,28,29,30]. It is often said that age increase leads to physiological changes that promote the onset of obesity and MS, often linked to a prothrombotic and pro-inflammatory state [12,30,31,32].

Regarding the frequency of MS components, reduced HDL cholesterol had a higher prevalence, followed by increased waist circumference and hypertriglyceridemia. It is acceptable to reflect on nutrition practices of PHC nursing professionals since they work in a context of precariousness, unhealthy conditions, long working hours, pressure for productivity, unfavorable safety conditions, and inadequate control over work and rest [33]. Among the functions of HDL cholesterol, we emphasize the contribution to the protection of the vascular bed against atherogenesis.Thus, it is plausible to avoid sources of polyunsaturated fatty and trans fatty acids in large quantity because they reduce HDL cholesterol levels [34].

Regular practice of physical activity is an auxiliary measure for preventing and controlling MS, which promotes a reduction in plasma triglyceride levels, blood pressure, waist circumference, glycemia and increased HDL cholesterol levels [31,35,36]. A cross-sectional study conducted in China indicates that consumption of alcoholic beverages increases the HDL cholesterol level, although excessive consumption raises blood pressure and the waist circumference level and, as a result, leads to MS [37].

Despite some similarities in Tsai’s findings; Cheng; Lai [23] and Felipe-de-Melo et al. [24], it is worth pointing out important methodological differences that suggest caution in comparisons: (i) the referred studies evaluated MS based on secondary data; (ii) they did not use variables related to labor conditions, and the scope of both was industrial environments; (iii) the association measurement adopted by the authors was odds ratio, which is not indicated for a cross-sectional study design, since discrepancies may be generated in the analyses [22]; (iv) Felipe-de-Melo et al. [24] did not measure waist circumference, but body mass index (BMI), and maybe they underestimated or overestimated the data.

The diverse spatial distribution of SM varied from 13.8% (São Francisco Valley Mesoregion) to 31.9% (Metropolitan Mesoregion of Salvador). Thus, studies per mesoregion are necessary to understand the differences related to behavioral/cultural and work-related determinants for SM. Thus, a systematic review of the literature has pointed out that obesity and risk factors for cardiovascular diseases in South America are mainly related to the effects of ethnicity and avoidable lifestyle and risk conditions, mostly in urban centers, where non-communicable chronic diseases were related to these factors [38].

Acanthosis nigricans is an easily identifiable skin lesion, characterized by thickening, hyperpigmentation and accentuation of the skin lines, having a rough and velvety appearance at the affected site and it is associated with insulin resistance. Insulin resistance is the most likely pathophysiological mechanism in MS [39]. Acanthosis nigricans was the most predictive variable associated with MS. In the bivariate analysis, insulin resistance increased by more than three times the occurrence of MS among nursing professionals. These two points are attested with the data found in the aforementioned research.

Being a nursing technician was associated with MS. In the investigated literature, no mechanism was found to explain such a finding, even though female nursing technicians present a higher level of occupational stress [40]. There is evidence that occupational stress is a risk factor for MS [33,41,42]. Burnout syndrome is a phenomenon initiated by chronic stress at work and reaches workers who have daily contact with people, making them less sensitive to environmental and labor issues [43]. Studies have pointed out an association between burnout syndrome and the MS components [35,44,45,46,47], however, no studies were found to associate burnout syndrome and MS.

The methodological standard used in this study confirmed the robustness of the results presented. In addition, the goodness of fit test and the area under the ROC curve ratified that the model has a reasonable predictive capacity. However, the following limitations should be pointed out: the impossibility of establishing a causal relationship between exposure variables and MS; susceptibility to biases, such as those related to the healthy worker; and memory, since some instruments used in the data collection had self-reported questions. In view of those situations and based on the transversal design applied in this study, some associations can be assumed. Between the independent variables and the outcome, the response rate obtained was 94.1%, which is an acceptable value for population-based studies [48]. In addition, high prevalence rates of exposure variables were observed, which is in line with the literature [8,24,27,33], even though these rates presented are underestimated.

## 5. Conclusions

A high MS prevalence was found among nursing professionals from the state of Bahia, Brazil. The factors associated with MS were physical inactivity, alcohol consumption, acanthosis nigricans, burnout syndrome, age greater than 36 years and being a nursing technician.

These results show and confirm the evidence presented in the literature on the biological plausibility of the association between work environment, occupational stress mechanisms and the presence of endocrine and cardiovascular diseases. In order to minimize the presence of MS, therefore, some interventions are necessary in the PHC environment, such as: improvement of working conditions, monitoring of worker health and safety with diagnostic procedures and medical referrals, as well as diet and physical activity programs. Future research should be developed in multiple health sectors to identify other exposure factors and stimulate the adoption of health and safety policies to the Brazilian nursing worker.

## Figures and Tables

**Table 1:** Prevalence of metabolic syndrome according to sociodemographic and labor variables in Primary Health Care Nursing Professionals, Bahia, Brazil, 2018, (*n* = 1111).

Variables	*n* (%)	Metabolic Syndrome (*n* = 1111) ^a^		
		P (%) ^b^	PR ^c^ (CI) ^d^	*p*-Value ^e^
Sex (*n* = 1125)				
Male	136 (12.1)	40 (29.4)	1.00	
Female	989 (87.9)	231 (23.7)	0.81 (0.61–1.07)	0.14
Age (*n* = 1125)				
Up to 35 years old	587 (52.2)	110 (18.9)	1.00	
36 years old or older	538 (47.8)	161 (30.2)	1.59 (1.29–1.97)	<0.01 *
Profession (*n* = 1125)				
Nurse	455 (40.4)	80 (17.7)	1.00	
Nursing Technician	670 (59.6)	191 (28.9)	1.64 (1.29–2.07)	<0.01 *
Race (*n* = 1098) ^a^				
Non-Black People	246 (22.4)	48 (19.5)	1.00	
Black People	852 (77.6)	220 (26.3)	1.35 (1.02–1.78)	0.03 *
Satisfaction with current occupation (*n* = 1125)				
Yes	987 (87.7)	233 (23.9)	1.00	
No	138 (12.3)	38 (27.7)	1.16 (0.87–1.55)	0.33
Occupation time in PHC (*n* = 1125)				
Up to 4 years old	555 (49.3)	109 (19.7)	1.00	
5 years old or older	570 (50.7)	162 (29.0)	1.47 (1.19–1.82)	<0.01 *
Night shift (*n* = 1125)				
No	894 (79.5)	206 (23.3)	1.00	
Yes	231 (20.5)	65 (28.6)	1.23 (0.97–1.56)	0.09
Family income (*n* = 1125)				
Up to two minimum wages	523 (46.5)	141 (27.5)	1.27 (1.03–1.56)	0.02 *
three or more minimum wages	602 (53.5)	130 (21.7)	1.00	
Economic situation (*n* = 1125)				
Satisfied	573 (50.9)	124 (22.1)	1.00	
Dissatisfied	552 (49.1)	147 (26.8)	1.21 (0.99–1.49)	0.07
Work bond (*n* = 1125)				
Stable	866 (77)	220 (25.8)	1.00	
Precarious	259 (23)	51 (19.8)	0.77 (0.58–1.01)	0.05 *
Submitted to work-related aggression (*n* = 1225)				
No	751 (66.8)	167 (22.6)	1.00	
Yes	374 (33.2)	104 (28.0)	1.24 (1.01–1.53)	0.04 *
Rest break (*n* = 1125)				
Yes	672 (59.7)	143 (21.7)	1.00	
No	453 (40.3)	128 (28.3)	1.30 (1.06–1.59)	0.01 *
Work conditions (*n* = 1125)				
Satisfactory	698 (62.0)	162 (23.6)	1.00	
Precarious	427 (38.0)	109 (25.7)	1.09 (0.88–1.34)	0.44

^a^ variables with missing data; ^b^ P: prevalence of outcome between exposed and non-exposed; ^c^ RP: gross prevalence ratio; ^d^ CI: 95% confidence intervals; ^e^ *p*-value: Pearson’s chi square test; * Statistical significance.

**Table 2:** Prevalence of metabolic syndrome according to lifestyle and human biology variables in primary health care nursing professionals, Bahia, Brazil, 2018, (*n* = 1111).

Variables	*n* (%)	Metabolic Syndrome (*n* = 1111) ^a^		
		P (%) ^b^	PRc (CI) ^d^	*p* value ^e^
Quality of life (*n* = 1125)				
Good	836 (74.3)	189 (22.9)	1.00	
Poor	289 (25.7)	82 (28.4)	1.23 (0.99–1.54)	0.07
Polycystic ovary (*n* = 964) ^a^				
No	807 (83.7)	192 (24.2)	1.00	
Yes	157 (16.3)	34 (21.7)	0.89 (0.65–1.23)	0.49
Psychiatric follow-up (*n* = 1111) ^a^				
No	728 (65.5)	169 (23.6)	1.00	
Yes	383 (34.5)	96 (25.3)	1.07 (0.86–1.33)	0.53
Practice of physical activities (*n* = 1125)				
Yes	639 (56.8)	130 (20.6)	1.00	
No	486 (43.2)	141 (29.3)	1.42 (1.15–1.75)	<0.01 *
Smoking (*n* = 1125)				
No	992 (88.2)	228 (23.3)	1.00	
Yes	133 (11.8)	43 (32.3)	1.39 (1.06–1.82)	0.02 *
Consumption of alcoholic beverage (*n* = 1125)				
No	1083 (96.3)	255 (23.8)	1.00	
Yes	42 (3.7)	16 (39.0)	1.64 (1.10–2.44)	0.03 *
Last medical consultation (*n* = 1125)				
Less than 12 months	924 (82.1)	222 (24.3)	1.00	
More than 12 months	201 (17.9)	49 (24.7)	1.02 (0.78–1.33)	0.89
Presence of periodontitis (*n* = 848) ^a^				
No	740 (87.3)	165 (22.6)	1.00	
Yes	108 (12.7)	27 (25.0)	1.11 (0.78–1.57)	0.58
Acanthosis Nigerians (*n* = 1125)				
No	1067 (94.8)	227 (21.6)	1.00	
Yes	58 (5.2)	44 (75.9)	3.52 (2.92–4.24)	<0.01 *
Non-alcoholic fatty liver disease (*n* = 1073) ^a^				
No	1040 (96.9)	242 (23.6)	1.00	
Yes	33 (3.1)	18 (54.5)	2.31 (1.66–3.22)	<0.01 *
Obstructive sleep apnea (*n* = 1076) ^a^				
No	967 (89.9)	212 (22.2)	1.00	
Yes	109 (10.1)	50 (45.9)	2.06 (1.63–2.61)	<0.01 *
Insulin resistance (*n* = 132) ^a^				
No	103 (78.0)	17 (16.5)	1.00	
Yes	29 (22.0)	17 (58.6)	3.55 (2.09–6.04)	<0.01 *
Burnout syndrome (*n* = 1121) ^a^				
No	916 (81.7)	192 (21.3)	1.00	
Yes	205 (18.3)	78 (38.1)	1.79 (1.44–2.22)	<0.01 *

^a^ variables with missing data; ^b^ P: prevalence of outcome between exposed and non-exposed; ^c^ RP: gross prevalence ratio; ^d^ CI: 95% confidence intervals; ^e^ *p*-value: Pearson’s chi square test; * Statistical significance.

**Table 3:** Factors associated with metabolic syndrome in primary health care nursing professionals, obtained by multivariate analysis.

Factors Associated with MS *	PR_adjusted_	CI (95%)
Physical activity	1.25	(1.02–1.53)
Consumption of alcoholic beverage	1.84	(1.22–2.77)
Acanthosis nigricans	3.23	(2.65–3.92)
Burnout syndrome	1.45	(1.17–1.81)
Age	1.37	(1.12–1.69)
Profession	1.43	(1.14–1.80)
Area under the ROC Curve	0.70	
Goodness-of-fit test ^¥^	0.62	

* Adjusted by smoking; ^¥^ Hosmer-Lemershow.
